# Nurse turnover and perceived causes and consequences: a preliminary study at private hospitals in Indonesia

**DOI:** 10.1186/s12912-018-0317-8

**Published:** 2018-12-19

**Authors:** Aryo Dewanto, Viera Wardhani

**Affiliations:** Postgraduate Program in Hospital Management, Faculty of Medicine, Universitas Brawijaya, Malang, Indonesia

**Keywords:** Nurse turnover, Private hospital, Causes, And consequences

## Abstract

**Background:**

Despite the inevitable growing rate of nurse turnover worldwide and its consequences, limited empirical data has been published in Indonesia. This study aims to describe the nurse turnover pattern at private hospitals, its causes and consequences as perceived by the hospitals’ managers.

**Methods:**

A survey method was used to obtain secondary and primary data from five private general hospitals in three administrative regions in East Java, Indonesia. The data of nurse turnover and demographic characteristics were collected. Mann Whitney test and relative risk analysis was performed to explore the role of nurse characteristics on nurses’ decision to leave their job. To explore the causes and consequences of nurse turnover, an online survey was conducted to twelve hospital managers. The data was then classified based on similar themes.

**Results:**

The data show that nurse turnover is between 12 and 34%. Being up to thirty years old, single, and having worked in the hospital up to three years significantly increase the risk of turnover. Personal reasons, external attractions and unsuitable working conditions are the three common nurse turnover reasons revealed by hospital managers. Hospital managers admitted that nurse turnover disturbs hospital operations, further impacting the hospital’s revenue and costs.

**Conclusions:**

The nurse turnover is higher than the acceptable level which is significantly predicted by age, marital status and job tenure. Further research is needed to develop nurse retention strategy in their early years of employment, based on the nurse’s point of view.

**Electronic supplementary material:**

The online version of this article (10.1186/s12912-018-0317-8) contains supplementary material, which is available to authorized users.

## Background

Nurse turnover is a rapidly-growing human resource problem currently affecting the healthcare sector worldwide. The rate across the world is considered high, ranging from 15 to 44% [1–3]. Limited studies published in Indonesian Journal showed that several private hospitals reveal figures similar to international literature, ranging between 13 and 35% [4–6]. These figures do not only show the high rate of nurse turnover worldwide, but also illustrate the wide-ranging values of known data. While even the lowest rate (15–18%) will lead to substantial financial and quality loss [1, 3], the highest rate is believed to cause many severe organizational consequences.

In the Indonesian context, managing nurse turnover in private hospitals is of the utmost importance because of the increasing role of the private sector in the Indonesian healthcare system. This increasing role is exemplified by the rapid growth of private hospitals (22%), a much higher figure than the 8% rise in government-owned institutions, and contributes to an overall higher proportion of private hospitals (60%) [7]. Consequently, the impact of high nurse turnover in private hospitals will not only affect the quality of care in these institutions but also have a considerable impact on the overall national healthcare system performance [8].

Despite the acknowledged importance of managing nurse turnover in private hospitals, the precise turnover rate and studies on its causes and consequences in Indonesia are not well documented. Much international literature has revealed the consequences of nurse turnover in health institutions in western countries [9–11], but limited empirical data has been published regarding nurse turnover in developing countries including Indonesia. As the developing countries, Indonesia encounters outstanding challenges in the field of health human resources, including shortages and maldistribution [12] that might lead to different turnover pattern, causes and consequences. The development of a prompt intervention to overcome turnover requires adequate support of data, resulting in the need for further investigation into this issue. This study is a preliminary survey aiming to describe the nurse turnover pattern at private hospitals, and its causes and consequences as perceived by the hospitals’ managers.

## Methods

A survey was performed to investigate the nurse turnover rate and its pattern, and further explore to its causes and consequences. The rate and pattern of nurse turnover were calculated based on a set of raw data. The data, provided by human resource department of five participating hospitals, contain sex, age, marital status, and duration and status of employment of 515 nurses who leave and stay in hospital in a one-year period. The participating hospitals represent 23.6% of the hospital bed capacity that serves a population of 3.4 million across three administrative regions which are Malang City, Malang Regency, and Batu City in East Java, Indonesia. The annual turnover rate was calculated by dividing the number of resigned nurses over a year with the average number of nurses over the same period and multiplied by 100 [13]. Mann Withney test and relative risk analysis were performed in this study to describe the role of demographic characteristics on the turnover decision.

Furthermore, to explore the causes and consequences of nurse turnover, an online survey that consist of five open questions (Additional file 1) was performed to obtain qualitative data. The survey was distributed to alumni affiliated to private hospital in Malang Raya through alumni’s social media group of Hospital Management Post-Graduate Program, Medical Faculty, Brawijaya University. The group consists of 127 members who work in various health institutions. Twelve (of 20 members affiliated to private hospitals in Malang Raya, and represent) responded to the survey (a 60% response rate). The respondents represent 30% of total number of private hospitals in Malang Raya. Through this survey, the causes and consequences of nurse turnover as perceived by the hospital managers were able to explore. In the survey, to explore the reason of nurse turnover they were asked about what were the common reason of nurses when they decide to leave the hospital. The managers were further asked about their opinion about the effects of nurse turnover on patients, remained nurses, doctors, and their hospital. The data were classified into similar themes of both causes and consequences.

## Results

### Nurse turnover rate

Overall data indicate that nurse turnover in private hospitals in Malang Raya shows a rate higher than 10%, with a broad range of between 12.78 and 34.15% (Table 1). Large hospitals, which have a number of beds and nurses higher than 100 (in this study, hospitals 1 and 2), show a lower turnover rate compared to small hospitals.

**Table 1:** Nurse turnover rate in five private general hospitals in Malang Raya in 2016

Hospital	Location	No. of Beds	No. of Nurses	Turnover Rate
Hospital 1	Malang City	135	103	14.02%
Hospital 2	Malang Regency	78	64	34.15%
Hospital 3	Malang Regency	220	198	12.78%
Hospital 4	Batu City	37	30	16.95%
Hospital 5	Malang Regency	53	43	25.64%
Total		525	438	

Source: Data of hospital human resource department (2016)

### Role of demographic characteristic on turnover decision

Our finding (Table 2) shows that of the total nurses (515 persons), most (67.57%) are female, and the majority (80.78%) are aged 30 years and under, with the largest proportion aged 25 to 30 years a (46.80%). Nurses who have worked three years or less have the largest total proportion (62.72%). More than half of the nurses are single (51.46%), and most nurses (80.78%) have been working since graduating from nursing education (new graduate / first job). Nurses who decided to leave the hospital are mostly women (62.3%), single (67.5%), and new graduates (76.6%). Most of them are up to 30 years old (94.8%) and have worked for not more than three years (93.5%).

**Table 2:** Nurse characteristics in five private general hospital in Malang Raya in 2016

Characteristics	Leave the hospital		Stay at the hospital		Total	
	Frequency	Percentage	Frequency	Percentage	Frequency	Percentage
	(*n* = 77)		(*n* = 438)		(*n* = 515)	
Sex						
·Female	48	62.30%	300	68.50%	348	67.57%
·Male	29	37.70%	138	31.50%	167	32.43%
Age						
·Up to 25 years old	40	51.90%	135	30.80%	175	33.98%
·More than 25–30-years-old	33	42.90%	208	47.50%	241	46.80%
·More than 30–35-years-old	4	5.20%	65	14.80%	69	13.40%
·More than 35-year-old	0	0.00%	30	6.80%	30	5.83%
Have worked for						
·Up to 1 year	36	46.80%	63	14.40%	99	19.22%
·More than 1 year – 2 years	22	28.60%	149	34.00%	171	33.20%
·More than 2 years – 3 years	14	18.20%	39	8.90%	53	10.29%
·More than 3 years	5	6.50%	187	42.70%	192	37.28%
Marital status						
·Married	25	32.50%	225	51.50%	250	48.54%
·Single	52	67.50%	213	48.60%	265	51.46%
Job status						
·First job (newly graduated)	59	76.60%	357	81.50%	416	80.78%
·Have had previous job	18	23.40%	81	18.50%	99	19.22%

Source: Data of hospital human resource department (2016)

The Mann Whitney test results show that marital status (U = 13,675.5, *p* = 0.002), age group (U = 12,089, *p* = 0.000), and length of time in the workplace (U = 9122.5, p = 0.000) were significantly related to turnover decisions, while sex, and status when starting work (newly graduated or experienced) were not.

To explain the tendency of demographic characteristics relating to the nurses’ turnover decision, we calculate the odds ratios and relative risk (Table 3). Before calculating relative risk and odds ratios, the age group and work period are re-classified by grouping each into two further categories, which are those aged up to 30 years and those aged above 30 years, and a work period of up to 3 years and a work period greater than 3 years.

**Table 3:** Relative risk of nurses’ decision to leave or stay in five private general hospitals in Malang Raya in 2016

Characteristics	Decision		Risk	Relative Risk
	Leave	Stay		
Sex				
·Female	48	300	0.14	0.79
·Male	29	138	0.17	1.26
Age				
·Up to 30	73	343	0.18	4.34
·More than 30	4	95	0.04	0.23
Have worked for				
·Up to 3 years	72	251	0.22	8.56
·More than 3 years	5	187	0.03	0.12
Marital status				
·Married	25	225	0.10	0.51
·Single	52	213	0.20	1.96
Job status				
·First job (newly graduated)	59	357	0.14	0.78
·Have had previous job	18	81	0.18	1.28

Source: Data of hospital human resource department (2016)

The relative risk result (Table 3) reveal that marital status, age, and duration of work are all related to nurses’ decision to leave a hospital. Being single, aged up to 30 years, and having worked in hospitals for up to 3 years are the risk factors of nurse turnover. The risk of leaving a hospital is almost twice as high for single nurses compared to those who are married (rr 1.96), more than four times as high for nurses aged up to 30 compared to those aged over 30 (rr 4.34), more than eight times as high for nurses who have worked up to 3 years compared to those who have worked for more than 3 years (rr = 8.56).

### Nurse’s reasons for leaving their job

**Fig. 1:**
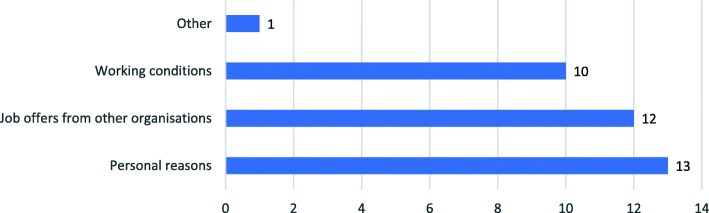
Causes of nurse turnover

The results of the online survey (Fig. 1) of the hospital managers show that reasons for nurses quitting their job are personal reasons (36%), accepting an offer from another organization (33%), working conditions (10%), and other reasons (3%).

Personal reasons are the most commonly given for nurses’ resignation, which vary from following their spouse (husband or wife) or family, getting married, having children, following a pregnancy program, and continuing their education. Accepting job offers from other organizations (government or private institutions) is the second most common reason for nurses leaving their jobs. Another common reason is working conditions, which include salary issues, uncomfortable work environment, lack of appreciation, communication barriers, and so on. The more detailed reasons are presented in Table 4.

**Table 4:** Nurses’ reasons for leaving their job as perceived by hospital managers

Reasons to leave the job	Frequency	Total	Percentage
Personal reason:		13	36.11%
• Follow spouse (husband or wife) or family, get married, do a pregnancy programme	10		
• Continue their study	3		
Accepting offer from other organisations:		12	33.33%
• Government institutions	7		
• Other hospitals	5		
Working condition		10	27.78%
• Salary	4		
• Uncomfortable working environment	2		
• Less recognition and reward	1		
• Feeling unsecure	1		
• Co-worker’s influence	1		
• Communication gap between hospital management and nurses	1		
Other reasons:		1	2.78%
• Not renewing the contract	1		

### Consequences of nurse turnover

The hospital managers state that nurse turnover disrupts hospital service operations (to diverse degrees ranging from fairly disruptive to very disruptive), and only three respondents state that nurse turnover is not disruptive. Respondents who state that turnover does not disturb their hospital operations mention that the turnover rate in their hospital is below 10%, and they have prepared several steps anticipating nurse turnover. However, almost all respondents state that nurse turnover negatively affects patients, nurses, doctors, and hospitals (Table 5).

**Table 5:** Consequences of nurse turnover in five private general hospitals in Malang Raya as perceived by hospital managers

Consequences on:			
Patient	Nurse	Doctor	Hospital
1. Patients perceive that nurses are not professional (because of the lack of skill) 2. Patients’ trust and satisfaction decreases 3. Patients’ complaints about nursing services increase	1. Senior nurses complain about higher workload because of nurse shortage 2. Nurses need to re-adapt and build new relationships, trust, teamwork 3. Senior nurses’ workload increases due to the new nurses’ adaptation process 4. Nurses feel reluctant to repeatedly teach and adapt to the new nurses	1. Doctors perceive that nurses lack skill 2. Nurses’ lack of skill disrupts service delivery to patients 3. Doctors feel unwilling to teach and adapt to the new nurses repeatedly 4. Nurse turnover increases Doctors’ complaints	1. Nurse turnover disturbs hospital services 2. Nurse turnover disturbs staffing practices 3. Nurse turnover leads to challenges in recruiting the replacement 4. Nurse turnover disturbs managerial processes 5. Nurse turnover increases hospital costs and disturbs hospital revenue

Respondents explained that nurse turnover disturbs hospital services, staffing processes, managerial processes and hospital revenue, and increases costs. The hospital managers revealed that the nurse turnover cause increase cost, such as recruitment and training cost for newly employed nurses. Nurse turnover negatively affects hospital services because new or replacement nurses’ skills do not meet the expected standards. New nursing staff need an adaptation and orientation process. Also, the service is likely to be less than optimal because of communication barriers between senior and new nurses, or between new nurses and other profession. Moreover, nurse turnover causes hospital staffing problems such as the loss of experienced and trained nurses, nurse shortages, and an imbalanced composition of new and remaining nurses. Those staffing problems cause difficulty in arranging work and setting schedules, necessitating increased over-time, and finally lowering the morale of existing nurses.

Furthermore, the respondents explain that their hospital has difficulty finding replacement nurses. This is not only because of the difficulties in replacing the quality of the nurses, but also because of the limited number of nurses. The unequal number of nurses and hospitals means hospitals are competing with each other to recruit new nurses. In addition to these problems, the process of preparing replacement nurses is time-consuming and requires many stages, starting from recruitment, orientation, and conducting required training. These challenges make hospitals experience nurse shortages across a period of time.

Regarding hospital finance, respondents explained that hospital revenue was also indirectly disrupted, due to the decreased work productivity of the new nurses in the adaptation stage. On the contrary, hospital expenses increase due to the costs of preparing new nurses, which range from recruitment, adaptation (mentoring) and training, to placement.

Aside from the impact on hospitals, hospital managers revealed that patients, nurses, and doctors are also impacted by nurse turnover. Patients acknowledge these frequent nurse replacements, as they feel they are being treated by a less competent new nurse, which leads to the patient’s decreased trust satisfaction, and increased complaints about nursing services. Additionally, the hospital manager explained that senior nurses feel unwilling to adopt an increased workload due to nurse shortages. Furthermore, the new nurse adaptation process generates several issues, such as difficulties in building trust, working relationships, and team work. Experienced nurses also feel that they are reluctant to repeatedly teach new nurses. Hospital managers also mention that nurse turnover increases doctors’ complaints about new nurses’ skills. Doctors further perceive that nurses’ lack of skill disrupts service delivery to patients. Like senior nurses, doctors become tired of adapting to the new nurses.

## Discussion

This research is a preliminary study that aims to provide an overview, rate, cause, and consequences of nurse turnover in Malang Raya. Many studies have been conducted in various western countries [9–11]. By using data from five private general hospitals in three administrative regions around Malang Raya in this study, we attempt to provide an overview of nurse turnover occurring in the Indonesian hospital.

Nurse turnover rate in the five private hospitals in Malang Raya ranges from 12 to 35%. This figure is similar to the results of some existing case studies in private hospitals in Indonesia [4–6]. The results of this study also show that the turnover rate is slightly lower compared to that worldwide, which is 15 to 44% [1–3]. Although the rates are slightly lower, turnover rates for most of the selected hospitals approach or even exceed 15%. International literature states that turnover with the lowest range between 15 and 18% leads to an increase in hospital costs, and disruption to hospital service operations [1–3]. This indicates that the actual turnover rate at selected hospitals in Malang Raya approaches and exceeds the lowest range and may therefore result in a significant decrease in service quality, and increase in hospital costs.

Single nurses aged up to 30 years old and having worked for less than and up to three years have a higher tendency to leave their jobs. Older nurses who have had a longer employment at the organization are less likely to leave [10]. These three demographic characteristics appear to be related, as nurses who worked for less than and up to three years are commonly young and single. Young nurses find it easier to leave their jobs for several reasons, such as unmet expectations, a desire for stability, and the necessity to achieve work-family balance [14]. Numerous areas of dissatisfaction appear among young nurses who are related to job content, such as a lack of supervision, and an uncertain and erratic working schedule [14]. In addition, young nurses experience changes at stages of their lives that must be adjusted to their work, such as getting married, having a baby, and moving their place of residence. They may even have to opt to leave their role despite loving their job as a nurse.

The hospital managers reveal that nurses quit their job for several reasons, which are personal reasons, accepting an offer from another organization, and working conditions. Nurse’s personal reasons in leaving their jobs are revealed by the hospital managers as being in line with the young nurses’ reasons for leaving their job [14], namely major life changes, such as marriage, having children, following pregnancy programs and so on. In addition, nurses leave their current job because of the availability of job offers from other organization. In Indonesia, nurses attempt to pursue jobs in the government sector or the credible private sector, such as in big hospitals. Nurses will move to another organization to find the better job security provided by government or credible private organization. In this study, big private hospitals may be considered as credible organization (as indicated by their smaller turnover rate). In the case of young nurses, transferring to other organization can be seen as a desire for stability and an adjustment to work-life balance as a result of changes in their life stages [14]. In addition to these reasons, nurses leave their job because of dissatisfaction with the work and the working conditions in their current employment [9–11, 14].

Respondents explained that nurse turnover disturbs hospital services, staffing practices, managerial processes and hospital revenue, and increases cost. Previous studies reveal similar results that the effects of nurse shortages are multiple; a high rate of turnover causes insufficient nursing staff, as well as bringing serious and wide-ranging organizational consequences. Turnover causes nurse shortages and disturbs nurse well-being, which in turn impacts patient care quality. Such shortages increase workload, overtime, and the utilization of temporary nurses [11, 15], and the consequential staff fatigue or low skill level of temporary nurses are likely to result in decreasing quality of care [1, 11, 15] and even an increase in patient safety incidents [15]. Additionally, overloading and overtime work can lower the morale of remaining nurses, which may in turn trigger further turnover [11]. Thus, these increase the burden on the hospital, which has ultimate responsibility for all costs incurred as a result of turnover [1–3, 11, 15].

### Practical implications

The results showed that the nurse turnover rates in private hospitals in Malang are considerably high, which may lead to negative consequences for hospitals and related parties. As personal reasons seem to be inevitable, thus the hospital manager should emphasize the strategies and optimize the solution regarding the two other causes: preventing resignations due to other organization’s job offers, and building conducive working conditions. For example, to address the unmet expectations of new nurses, HR departments should optimize the selection process so they can understand the prospective new nurse’s expectations and not promise them condition that cannot be attained.

### Limitation of the studies

The sample of this study may not randomly represent Malang Raya or Indonesia, but the characteristics of the figures from the sample are quite similar to the overall situation in Indonesia. Also, the causes and consequences are based on hospital managers’ perspectives and may not fully represent the underlying reasons, but at the same time they communicate the managers’ point of view as the prime stakeholder.

## Conclusions

The rate of nurse turnover in private hospitals revealed from our study is considered high, potentially resulting in negative impacts, and should be reduced.

Being single, less than and up to 30 years old, and having a working period of less than and up to three years are factors likely to play role in nurse turnover decisions, suggesting that a potential way to reduce turnover is to retain nurses in an organization more than three years. Other nurse characteristics – of sex and nurses’ status at work (fresh graduates or inexperienced) – are not related to the turnover decision.

The three most common reasons for nurses leaving a hospital are personal reasons, job offers from hospitals or other organizations, and working conditions. Hospital management view nursing turnover as a disturbance to hospital services, staffing practices, managerial processes, and hospital revenue, which increases costs. Furthermore, nurses and doctors feel the immediate impact of nurse turnover.

The hospital managers’ point of view related to the causes and consequences of nurse turnover is a good entry point that needs further exploration from the nurse’s perspective to reveal its underlying reasons.

## Additional file

Additional file 1: Online Survey Questions (in Bahasa Indonesia and English). This file contains the online survey questions which are written in two version, i.e. original language (Bahasa Indonesia) and translated (English) version. (DOCX 14 kb)
