# Supplementary material for: Nurse turnover and perceived causes and consequences: a preliminary study at private hospitals in Indonesia
Source: BMC Nurs. 2018 Dec 19;17(Suppl 2):52. doi: 10.1186/s12912-018-0317-8 (PMC6299511; doi:10.1186/s12912-018-0317-8)
Supplement: Supplementary file 1 — Online Survey Questions (in Bahasa Indonesia and English). This file contains the online survey questions which are written in two version, i.e. original language (Bahasa Indonesia) and translated (English) version. (DOCX 14 kb) [file 12912_2018_317_MOESM1_ESM.docx]

**Additional file 1.** Online Survey Questions

**Original version (in Bahasa Indonesia)**

1. Jelaskan apakah turnover perawat mengganggu operasional rumah sakit anda!
2. Jelaskan penyebab perawat melakukan turnover di rumah sakit anda! (Tuliskan secara berurutan mulai dari penyebab yang paling sering diungkapkan oleh perawat)
3. Bagaimana dampak nyata turnover yang dirasakan oleh rumah sakit anda? (Tuliskan berurutan dari dampak yang dianggap paling berat)
4. Bagaimana dampak nyata turnover di rumah sakit anda bagi:
   1. Pasien
   2. Perawat
   3. Dokter
   4. Pihak lainnya (bila ada)
5. Apakah upaya yang sudah dilakukan oleh rumah sakit anda untuk mengurangi turnover perawat ini? (bila ada)

**Translated version (in English)**

1. Explain whether nurse turnover disturbs your hospital operations
2. Explain why nurses left from your hospital (Please write starting from the most commonly disclosed cause by nurses)
3. How do the actual impacts of nurse turnover in your hospital? (Please write starting from the hardest impacts)
4. How do the actual impacts of nurse turnover on:
   1. Patients,
   2. Nurses,
   3. Doctors,
   4. Other parties (if any)?
5. What efforts have your hospital taken to reduce this nurse turnover? (if any)
